# Peatland fires in Alaska will double by the end of the century

**DOI:** 10.1038/s41598-025-19682-4

**Published:** 2025-10-14

**Authors:** Mark Jason Lara, Roger Michaelides, Duncan Anderson, Wenqu Chen, Emma Catherine Hall, Caroline Ludden, Aiden Isaac Gittler Schore, Umakant Mishra, Sarah Nicole Scott

**Affiliations:** 1 Department of Plant Biology, University of Illinois, Urbana, IL USA; 2 Department of Geography, University of Illinois, Urbana, IL USA; 3 Department of Geography, Washington University at Saint Louis, Saint Louis, MO USA; 4 Computational Bio & Biophysics, Sandia National Laboratories, Livermore, CA USA; 5 Thermal/Fluids Science and Engineering, Sandia National Laboratories, Livermore, CA USA

**Keywords:** Ecology, Climate-change ecology, Environmental impact

## Abstract

During recent summers, warm and dry conditions have increased the occurrence of wildfires and potentially peat-fires across Alaska. Limitations in resolving the fine-scale distribution of peatlands and climate observations have constrained our ability to accurately predict peat-fire dynamics. Using a new high-resolution peatland map of Alaska, we evaluated the climate and environmental controls of past and future peat-fire activity. Ensemble machine learning models identified reduced soil moisture, higher temperatures, and evapotranspiration as key predictors of annual total burned peatland area (tenfold CV R^2^ = 0.62, RMSE = 221.1 km^2^). By the end of the twenty-first century, models forced with climate datasets from representative concentration pathways (RCPs) 4.5, 6.0, and 8.5 emission scenarios project a statewide doubling of burned peatlands (increasing 61–121%), with regional increases ranging from 25–165% in polar, 61–95% in boreal, and 102–106% in maritime ecoregions. These projections indicate that wildfires will progressively encroach further into organic-rich moist and wet peaty soils, potentially amplifying soil carbon release across Alaska.

**Supplementary Information:**

The online version contains supplementary material available at 10.1038/s41598-025-19682-4.

## Introduction

Peatlands cover 3% of the global land surface yet store 25% of the world’s soil organic carbon^1^. These organic-rich soils are widespread across permafrost regions, accounting for nearly 18% of the land surface and storing about 500 and 600 PgC^2–4^. As warmer and drier conditions become more prevalent across northern ecosystems, the vulnerability of peatland soils may increase, not only due to increased rate of aerobic respiration^5^, but also due to the susceptibility of peat-fires, as partially decayed organic matter becomes drier and more prone to peat-fires^6–9^. Peat-fires pose severe consequences for the global climate, potentially releasing up to 15% of annual anthropogenic emissions, mobilizing an estimated 0.35–1 GtCyr^−1^^10–12^, thereby accelerating climate warming ^13,14^.

Unlike flaming fires, peat-fires often smolder slowly within the soil matrix for weeks to months after ignition, consuming large volumes of soil carbon^12,15,16^. As dried organic matter is highly flammable, smoldering peat can persist even under prolonged periods of rainfall and snow cover^17,18^, continuing to deepen the depth of burn by several centimeters to decimeters^19,20^. However, the magnitude, duration, and spatial heterogeneity of peat combustion are typically modulated by peat moisture, storativity (i.e., physical moisture storage properties), porosity, and the variable capillary fringe^21^, which can create uneven pore structures that affect water content and act as barriers to peat smoldering^22^. As a result, warmer climatic conditions may not only increase the frequency of wildfires^23–25^, but also accelerate peat desiccation, raising the likelihood of peat ignition and consumption^6,26,27^. These challenges, combined with uncertainties in fine-scale mapping of peatland extents^28^, make forecasting future peat-fire dynamics highly uncertain and difficult to predict^20,29^.

**Fig. 1:**
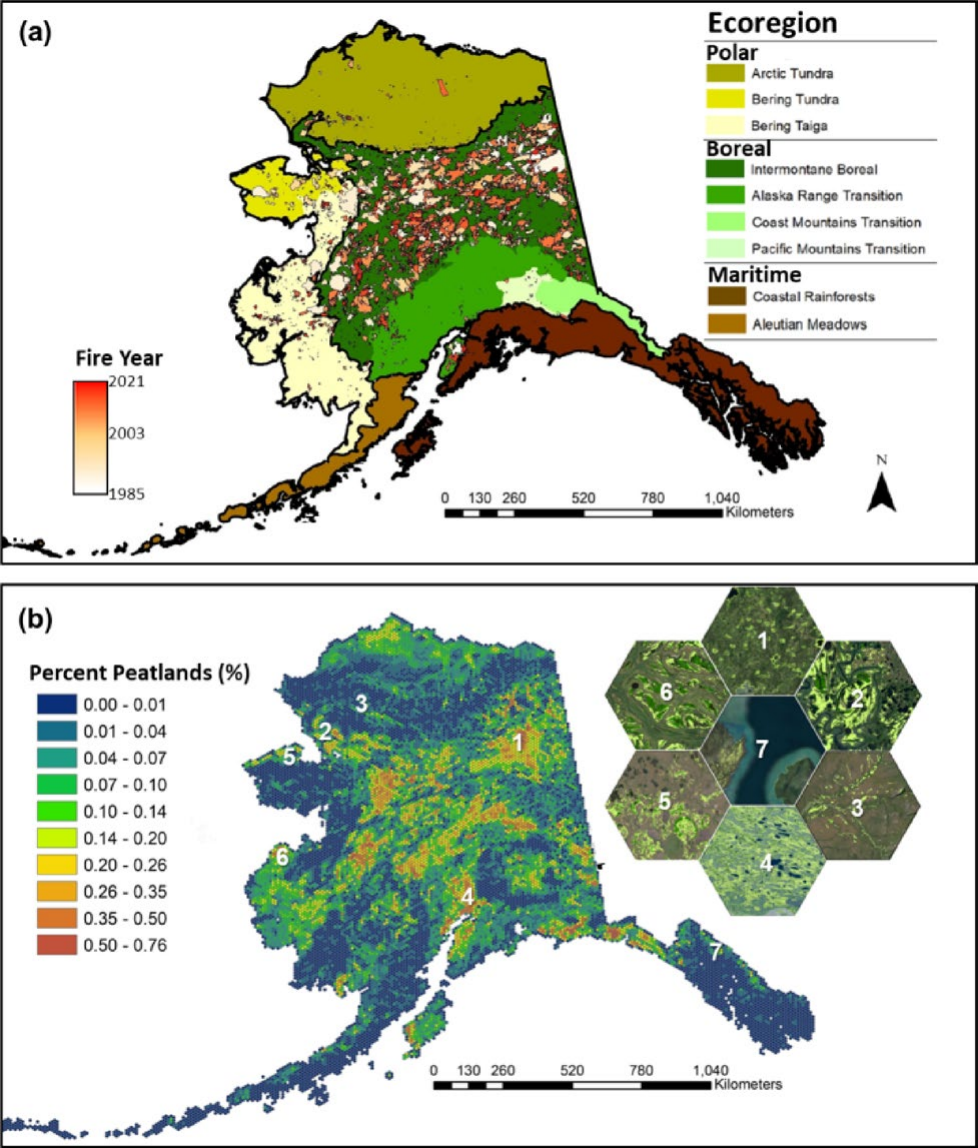
Spatial distribution of wildfires and peatlands across ecoregions of Alaska. Ecoregions level 1 (i.e., Polar, Boreal, and Maritime) and level 2 (e.g., Arctic tundra, Aleutian Meadows; panel a) are defined by Nowacki et al.^34^ while historical fire scars from the Alaska Interagency Coordination Center^35^, respectively. The high-resolution (20 m) data from the peatland extent map of Alaska was generated in Google Earth Engine using a combination of publicly available optical, radar, and elevation products ^31^ and visualized in ArcGIS version 10.8.1 (https://www.esri.com/) to estimate the percentage of peatlands within each 10 km tessellated pixel. Selected zoomed-in tessellated pixel examples from multiple ecoregions are presented in panel b, overlaid on satellite images obtained from © 2015–2022 Maxar Technologies (https://www.maxar.com/).

High-latitude peatlands have been challenging to detect and are poorly mapped^6,28,30^, leaving the past and projected impacts of peat-fires on regional to global carbon cycle processes uncertain. Detection is hindered by the fine-scale spatial heterogeneity of peatland moisture regimes, surface/subsurface hydrologic connectivity, vegetation composition and canopy architecture which can vary from meters to kilometers^31^. Coupling this biocomplexity with the stochastic nature of wildfire –which is projected to increase in frequency and severity^24,25,32^, makes it challenging to assess the vulnerability of peatlands to wildfire. As a result, our understanding of the factors that govern peat-fire dynamics across northern latitudes remains limited, contributing to uncertainties in future climate-carbon feedbacks^6^. In this study, we leverage the first wall-to-wall, fine-scale (20 m spatial resolution) peatland extent map of Alaska (~ 1.5 million km^2^)^31^ to assess how climate and environmental factors (via., ERA5-Land climate reanalysis data) influence the sensitivity of peatlands to burning under both historical (i.e., 1985–2022) and projected (i.e., 2090–2100) conditions. By integrating historical fire records and climate reanalysis data with new statewide peatland datasets^31,33^ we predict (1) the annual total area of peatland burned, and (2) the proportion of peatlands burned within fire scars across all ecoregions in Alaska (Fig. 1, Supplemental Fig. 1), using ensemble machine learning techniques. These findings provide new insights into the factors controlling, trajectories of, and implications for twenty-first century peat-fires across Alaska.

## Results

### Temporal variability in peat-fires

The total burned area (including both peatlands and non-peatlands), along with its standard error, varied notably across polar (697.1 ± 350.8 km^2^ yr^−1^), boreal (9,169.7 ± 4,565.7 km^2^ yr^−1^), and maritime ecoregions (20.6 ± 11.5 km^2^ yr^−1^). However, using both the AICC fire history data and the peatland extent map of Alaska, we estimate peatlands to constitute only a small portion of the total burned area, as indicated by the proportion of burned peatlands relative to the total burn area: 6.8% ± 1.3% in polar, 18.6% ± 1.3% in boreal, and only 3.5% ± 1.6% in maritime ecoregions. Consequently, the average annual burned peatland area across ecoregions was 34.3 ± 12.8 km^2^ in polar, 806.6 ± 172.1 km^2^ in boreal, and 1.6 ± 1.0 km^2^ in maritime ecoregions (Supplemental Fig. 2). After removing spatial autocorrelation using an autoregressive model, Spearman’s correlation coefficient showed a significant increase over time in the total peatland area burned (r = 0.32, *p*-value < 0.05) but not in the proportion of peatland area burned (r = 0.22, *p*-value = 0.18) in polar ecoregions. In polar ecoregions, the total peatland area burned increased nearly ninefold, from 11.4 to 98.5 km^2^ per year, while the proportion of burned peatlands tripled, increasing from 4.3 to 13.5% between the most recent decade (2013–2022) and the previous three decades (1985–2012). However, no other significant correlations with time were found in the other ecoregions.

**Fig. 2:**
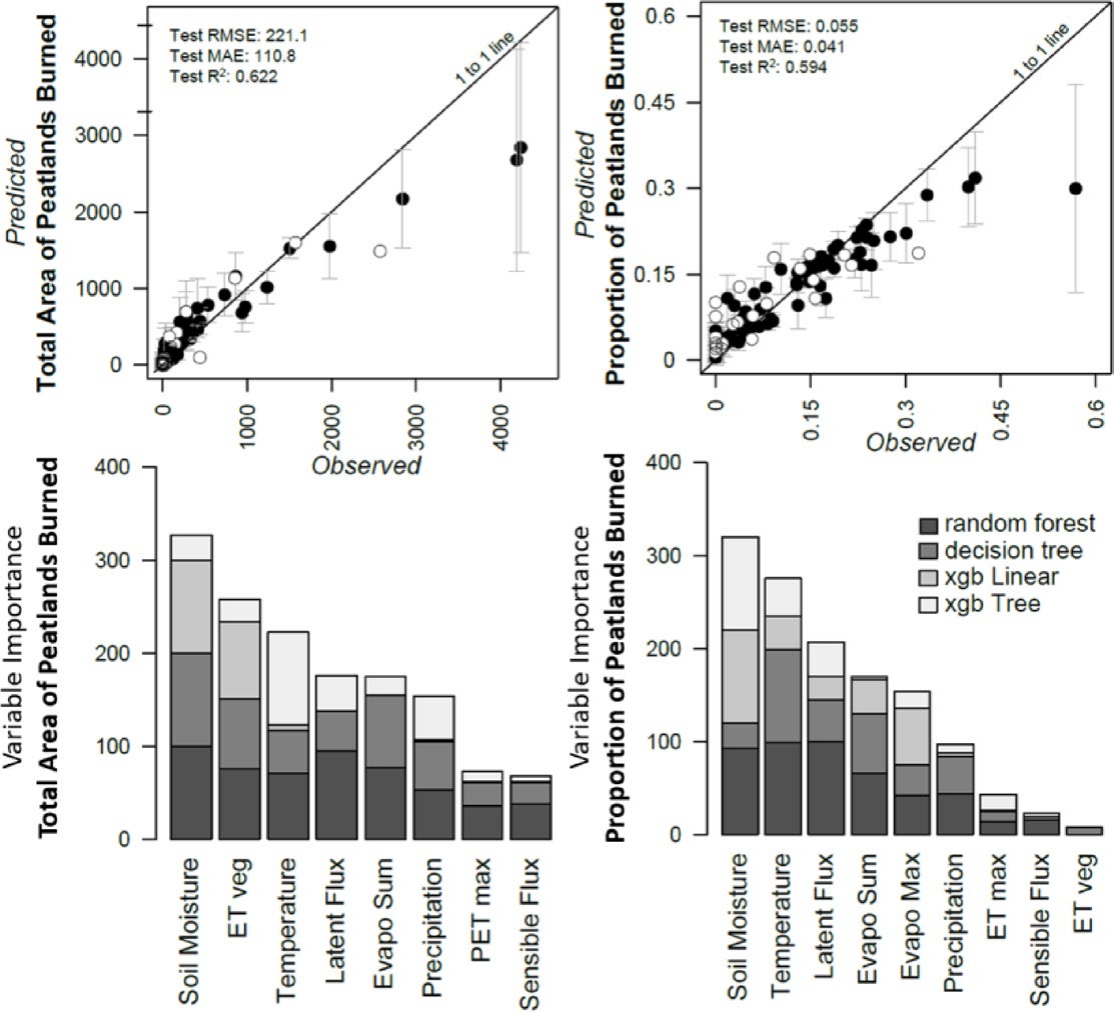
Ensemble models of the annual total area of peatlands burned (km^2^, left column) and annual proportion of peatlands burned (right column) between 1985 and 2022 across Alaska. Observed versus predicted plots display trained observations in black circles and testing observations in white circles. Error bars for observations are derived from the uncertainty among base and meta-models. Independent evaluation of ensemble performance was evaluated with RMSE, MAE, and tenfold CV R^2^. Variable importance plots (bottom row) display stacked bars of the most influential predictors among boosting and bagging models. See methods for variable descriptions.

### Peat-fire modeling

Ensemble models accurately predicted both the total area of peatlands burned and the proportion of peatlands burned across Alaska (Fig. 2). The model’s performance, evaluated by leave-one-out ten-fold cross-validation R^2^ and hold-out validation yielded RMSE and MAE values of 0.62, 221.1 km^2^, and 110.8 km^2^ (training: 0.93, 204.1 km^2^, and 127.5 km^2^) for the total area of peatlands burned, and 0.59, 5.5%, and 4.1% (training: 0.91, 3.0%, and 2.2%) for the proportion of peatlands burned, respectively. The relatively lower cross-validation R^2^ values in the testing versus training datasets may indicate slight overfitting; however, this discrepancy is attributed to the smaller sample size of testing datasets, which may not fully represent the range of values for the total area and proportion of peatland area burned. Notably the testing data was well within the model’s uncertainty bounds. To minimize overfitting, hyperparameters were tuned to reduce tree complexities and adjust parameters (e.g., minobs, min_child_weight) to increase pruning and produce simpler models (Supplemental Table 1). Soil moisture emerged as the most influential predictor for both peat-fire metrics in the ensemble- and base models (Fig. 2). However, the overall variable importance of predictors varied slightly between models. For example, the top five predictors for the total peatland area burned across all base models in the ensemble were soil moisture, evapotranspiration, temperature, latent heat flux, and the sum of evaporation. In contrast, the top five predictors for the proportion of peatland area burned were soil moisture, temperature, latent heat flux, sum of evaporation, and maximum evaporation. Partial dependency plots indicated that higher peat-fire activity (e.g., total area and proportion of peatlands burned) was associated with low precipitation, high evaporation, and warmer temperatures, while regionally-specific differences in soil moisture appeared to closely correspond with Alaska’s physiographic and climate gradients linked to more fire-prone landscapes.

**Fig. 3:**
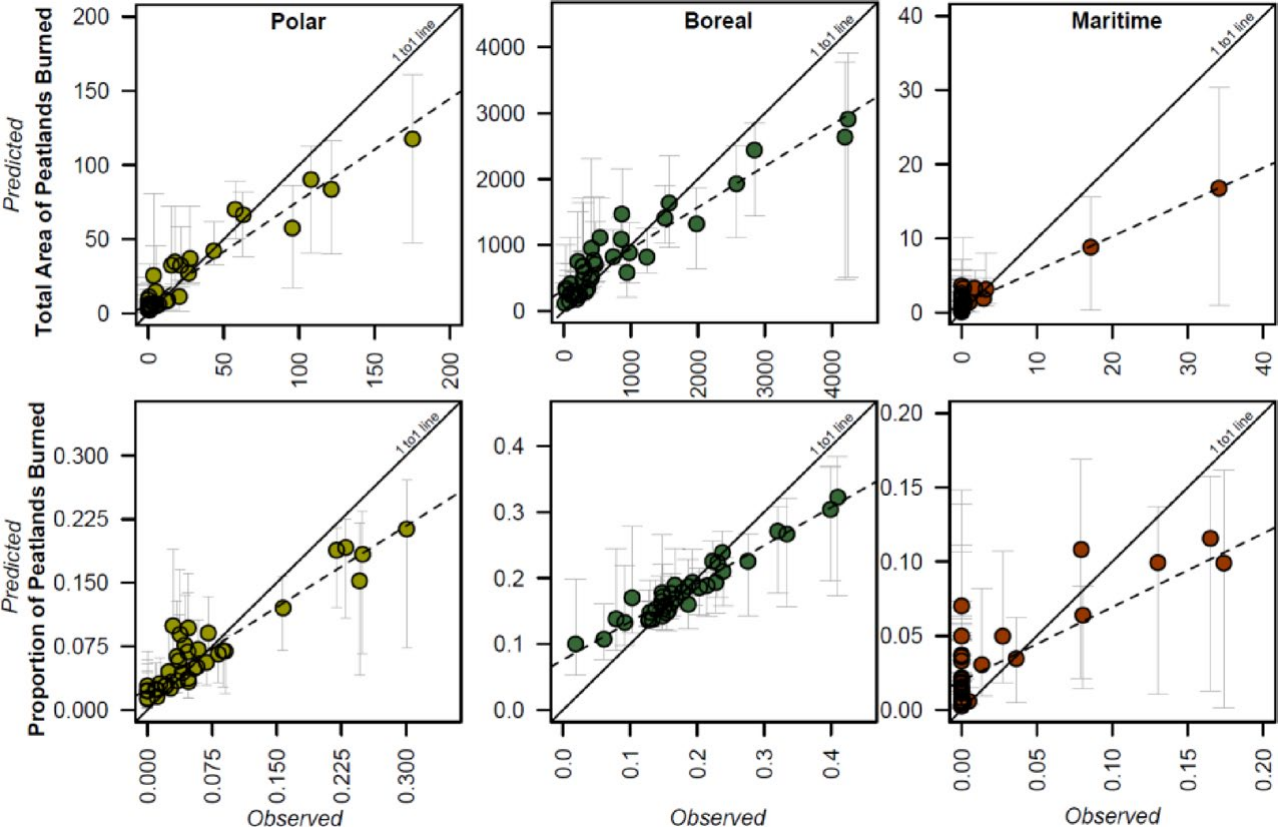
Ecoregion-specific random forest models for annual total peatland area burned (km^2^, top row) and annual proportion of peatlands burned (bottom row). Error bars represent 95% prediction intervals derived from bootstrap-based resampling with replacement.

Performance metrics demonstrated that ensemble models for both peat-fire metrics outperformed individual models, as indicated by lower RMSE and MAE values (Supplemental Table 2). Though ecoregion-specific random forest models well predicted the variability of peat-fire metrics over time (Fig. 3), the variable importances from random forest models varied by ecoregion (Supplemental Table 3); consistent with those identified in the ensemble models (Fig. 2). The tenfold cross-validation R^2^ and RMSE values for the total area of peatlands burned in polar, boreal, and maritime ecoregions were 0.77 and 25.7 km^2^, 0.59 and 853.1 km^2^, and 0.50 and 4.6 km^2^, respectively. For the proportion of peatlands burned, the tenfold cross-validation R^2^ and RMSE for polar, boreal, and maritime ecoregions were 0.46 and 6.1%, 0.43 and 7.6%, and 0.50 and 7.2%, respectively. In polar ecoregions, the key variables determining both the total peatland area burned and the proportion of peatlands burned were consistently the sum of evaporation, mean temperature, and the sum of latent heat flux (Supplemental Table 3). In boreal ecoregions, the most important variables varied: the total area of peatlands burned was primarily influenced by maximum evapotranspiration and soil moisture from deep soil layers, whereas the proportion of peatlands burned was best predicted by mean temperature, the sum of latent heat flux, soil moisture from shallow soil layers, and the sum of evaporation. In maritime ecoregions, mean temperature and latent heat flux were consistently the most important variables for predicting peat-fire metrics (Supplemental Table 3). Importantly, limited historical wildfire activity in maritime ecoregions (Supplemental Fig. 2) increased prediction uncertainty (e.g., Fig. 3), and as such, trends from this ecoregion must be interpreted conservatively.

**Fig. 4:**
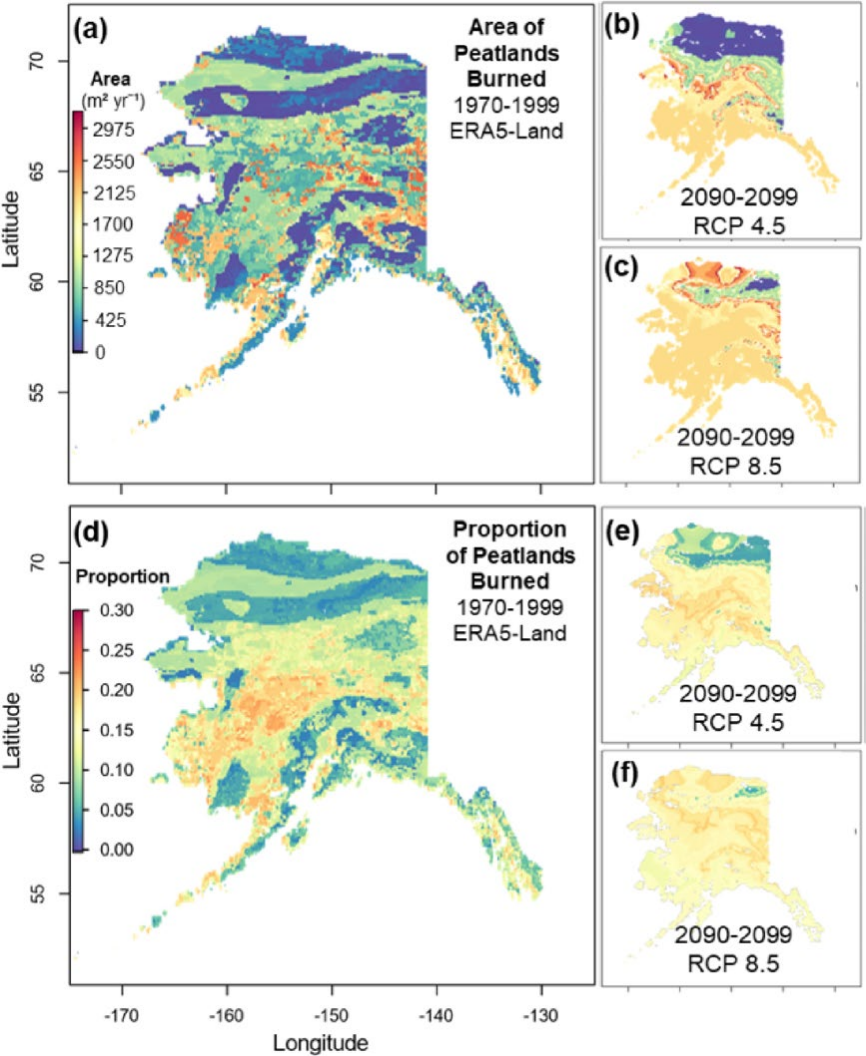
Ensemble model derived past (1970–1999 mean) and projected (2090–2099 mean) patterns of peat-fire metrics across Alaska. Historical forcing data were derived from ERA5-Land reanalysis data and projected data were derived from the top-5 performing CMIP5 models for Alaska^36^ for representative concentration pathways (RCP) 4.5, 6.0, and 8.5 (see Supplemental Fig. 4 for 6.0). Peat-fire models and associated geospatial layers were generated in R version 4.3.2 (https://www.r-project.org/) and visualized in ArcGIS version 10.8.1 (https://www.esri.com/).

**Fig. 5:**
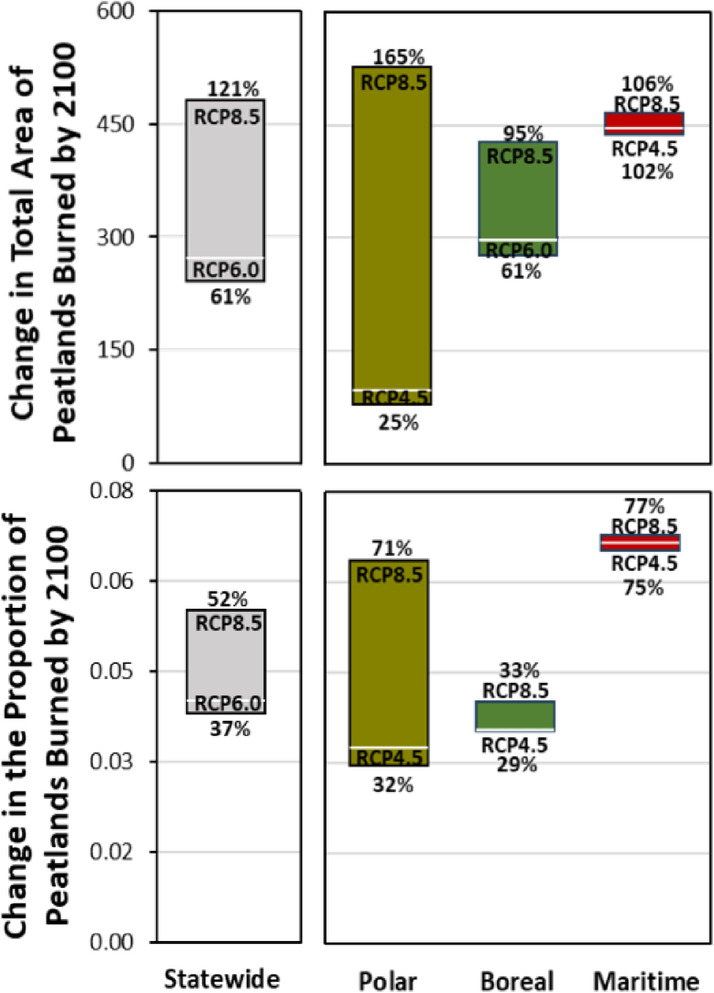
Simulated change in peat-fire metrics by the end of the twenty-first century. Ranges of uncertainty for statewide, polar, boreal, and maritime ecoregions correspond to climate uncertainties from Representative Concentration Pathways (RCPs) 8.5, 6.0, and 4.5, where the highest and lowest RCPs are labeled at the top and bottom of each range with corresponding percent change, respectively. White line within each uncertainty range represents the unlabeled RCP.

Ensemble models were used to simulate the likely twenty-first century change in peat-fire activity across Alaska. Modeled historical spatial patterns of both the total area of peatlands burned and the proportion of burned peatlands within fire scars, identified the highest peat-fire metrics in the Intermontane boreal forest and Bering tundra ecoregions (Fig. 4). Peat-fire model forecasting by 2090–2099 indicates that the total area of peatlands burned will more than double under RCP 8.5 climate scenarios with increases of 120.7%, relative to the historical mean baseline (1970–1999), whereas RCPs 4.5 and 6.0 increased 61.3% and 61.1%, respectively (Fig. 5, Supplemental Table 4). Although the projected changes in polar regions are relatively minimal in both burn metrics under RCPs 4.5 and 6.0, polar ecoregions become comparable to the expected changes in maritime and boreal ecoregions under an 8.5 scenario (Figs. 4, 5). However, the potential uncertainties in polar regions due to future emission scenarios are large, ranging from a 164.9% increase under RCP 8.5 to a 25% increase under RCP 4.5 (Supplemental Table 4). Nevertheless, simulations project that wildfires across Alaska will affect a greater proportion of peatlands within each fire scar in the future, with statewide increases ranging from 36.5 to 51.9% (Fig. 5, Supplemental Table 4).

## Discussion

Due to increasingly warmer and drier summer seasons across Alaska, wildfire activity has been extraordinarily high in both polar (i.e., 2015 and 2022) and boreal ecoregions (i.e., 2004, 2005, 2009, 2015, 2019, 2022; Supplemental Fig. 2). Consequently, observations of regional peat-fires have increased^19,37^. Our results indicate that peat-fires have significantly increased across polar ecoregions over the past ~ 40 years, with the most recent decade experiencing nearly nine times the total peatland area burned compared to the previous three decades. Ensemble models provide new quantitative evidence suggesting that as the climate continues to warm, increased evaporation and evapotranspiration will cause peatlands to dry, making them more susceptible to burning (Fig. 2). Climate projections indicate that these drought-like conditions will intensify by the end of the twenty-first century^38,39^, as our peat-fire simulations indicate the total area of burned peatlands will increase by 61–121%, intensifying at northern latitudes from RCP 4.5 to 8.5 with wildfires burning 37–52% more peatland area across Alaska (Figs. 4, 5), potentially leading to greater consumption of deep, carbon-rich soils.

Although the climatic and environmental controls identified by ensemble models well-represented statewide peat-fire processes, the specific controls varied among ecoregions and peat-fire metrics (Supplemental Table 3). This variation in controls among ecoregions is not surprising, as many types of peatlands are present across these ecoregions (e.g., fens, bogs, marshes, tundra meadows), each influencing their vulnerability to fire occurrence^20,40^ in distinct ways. Interestingly, we found that the controls on both peat-fire metrics (i.e., total peatland area burned and the proportion of peat area burned) were similar within polar and maritime ecoregions but diverged in boreal ecoregions. For example, in boreal ecoregions, the combined loss of peatland moisture from aboveground vegetation (i.e., evapotranspiration) and belowground sources (i.e., deep soil moisture) explained nearly all the variability in the total area of peatlands burned between 1985 and 2022. However, these factors explained little of the variability in the proportion of peatlands burned (Fig. 4, Supplemental Table 3). This discrepancy was likely due to a positive linear correlation between both peat-fire metrics in polar (r = 0.82) and maritime ecoregions (r = 0.87), but not in boreal ecoregions (r = − 0.03). This suggests that peat soils may be more homogenously distributed across polar and maritime ecoregions, where an increase in the total area burned corresponds with an increase in the total area of peatlands burned. However, this pattern does not hold in the more fire-prone boreal ecoregions, which exhibited a greater spatial heterogeneity across upland (i.e., coniferous and deciduous forests) and lowland ecosystems, leading to contrasting patterns in the total area of peatlands burned and the proportion of peatlands burned. For example, during the 2004 wildfire season, only 4240 km^2^ of peatlands burned of the estimated 26,863 km^2^ total burned area (~ 15.7%) across the Alaskan boreal forest, with most fires occurring in upland regions. Whereas, during the 2001 wildfire season, 358  km^2^ of peatlands burned of the estimated 873 km^2^ total burned area (~ 40.9%), likely a result of diverse boreal landscapes that form a mosaic of peatland and non-peatland landforms, including treed bogs, bogs, fens, marshes, swamps, palsas, permafrost plateaus, hummocks, and hollows, each varying in flammability^20^ and sensitivity to climatic conditions and ignition^41^. Though there are many physical and meteorological fine-scale processes that influence fire behavior (e.g., wind speed)^42,43^ which were beyond the scope of our peat-fire modeling, this data-driven application aimed to represent the general spatial and temporal patterns that govern the trajectories of peat-fire dynamics across Alaska (Figs. 4 and 5).

Ensemble models support the idea that the susceptibility and resilience of northern peatlands to burning are explicitly linked with peatland hydrology. As peatland water tables decrease, our new estimates of wet and moist peat may better inform fire prediction models, especially as warming increases the desiccation of the top layers of previously saturated peat, making them more flammable and susceptible to smoldering and overwintering fires. Although recent remote sensing derived peat-fire indices^44^ may improve the detection of burned peat in future iterations of this analysis, our likely overestimation of burned peat within fire scars necessitates a cautious interpretation of our peat-fire metrics. We can approach this in two ways: (1) as an estimate of the burned peatland extent, acknowledging that the actual annual total area of peatlands burned will likely be proportional to the total area burned, and (2) as the proportion of burned peatlands, which can either be represented as a direct estimate of the ratio of the area of peatlands to non-peatlands that burned, or more conservatively as an indication over time of the fuel sources wildfires are consuming. For example, our analysis revealed positive trends in the proportion of peatlands burned in polar regions, suggesting that wildfires have increasingly encroached into terrain historically dominated by inundated peatlands (Supplemental Fig. 2), while statewide projections indicate that warmer temperatures and increased evaporation rates could lead to an increase in the proportion of peatlands versus non-peatlands burned by up to 121% in polar regions and 52% across Alaska by the end of the century (Fig. 5).

Projected increases in peat-fire activity have significant implications for the reduction of large peatland organic carbon stocks^2–4^. As the frequency and spatial extent of peat-fires increase, these slow-burning fires may consume nearly twice as much organic carbon as non-peat fires due to their prolonged smoldering at depth^18^. The loss of peat will be further magnified by the reduction or elimination of peatland water tables, as fires can propagate deeper, with carbon emissions potentially increasing up to nine-fold following complete drainage^26^. Additionally, as extreme summer temperatures increase, fire seasons will become longer and larger, facilitating more frequent and extensive peat-fires (even in polar ecoregions; e.g., Fig. 4). These fires may even re-emerge as overwintering or “*zombie*” fires^30,45,46^. Although the contribution of overwintering peat-fires to historical carbon losses has been relatively small, their potential impact is significant, with an estimated ~ 3.5 Tg of carbon emissions released between 2002 and 2018 in the Northwest Territories and Alaska, where 64% of these emissions occurred during a single warm and dry fire season^30^. Our analysis suggests that the projected increase in wildfires across Alaska^47,48^ will increase the proportion of peatlands burned (Figs. 4e and f, 5), likely increasing the frequency and impact of overwintering fires^30,49^.

As high-latitude peatlands become increasingly threatened by warmer and drier conditions, this research provides an important first step in quantifying fine-scale impacts on peatlands across Alaska. Our findings suggest that with continued warming, wildfires will progressively encroach into organic-rich peaty soils, leading to a gradual but significant release of ancient carbon into the atmosphere^49^. Nevertheless, the potential impact of peat-fires may be even greater than reported, as our analysis only included fires greater than 400 ha in size, excluding smaller fires that still account for ~ 19,500 ha yr^−1^ (0.17% of total area burned) and may hold ecological significance. With accelerating permafrost degradation^11,50,51^ and associated changes in subsurface hydrology^6,26^, these carbon-rich soils are expected to become increasingly vulnerable to drying, draining, and burning^46^.

While new high-resolution peatland datasets^31,33^ improve our ability to assess the region-specific sensitivity, vulnerability, and/or resilience of peatlands to drying and ignition, future research should integrate ongoing trajectories of landscape evolution^50,52–57^ , including shifts in peatland extent, burn severity, burn depth, and associated carbon losses. Importantly, as the number of fire seasons observed by spaceborne spectral and thermal sensors increase, there will be new opportunities to detect the fraction of burned peat within fire scars (e.g., peat-fire index^44^) improving the detection of both peat-fires and overwintering fires, while providing new insights into the timing of ignition, seasonal burn extent, and the re-emergence of fires. Ultimately, fine-to-meso scale applications will deliver actionable information necessary not only for quantifying historical losses of peatland carbon pools but also for improving projections of carbon emissions and smoke forecasting of particulate matter, carbon monoxide, nitrogen oxides, and other volatile organic compounds emitted during flaming and smoldering events across Alaska.

## Methods

### Ecoregions of Alaska

Alaska consists of three major ecoregions: the polar, boreal, and maritime ecoregions, comprising 25, 57, and 18% of the total land area, respectively^34^ (Fig. 1). Over the past 65 years, the state has experienced a warming trend of approximately 0.35 °C decade^−1^^58^. In the polar ecoregions, temperature and precipitation have increased by about 0.53 °C decade^−1^ and 3.2% decade^−1^, respectively (Ballinger et al.^58^). These regions are underlain by continuous to discontinuous permafrost and are covered by various mosses, sedges, and dwarf to low shrubs growing in moist-acidic soils^59,60^. In the boreal ecoregions, significant warming and drying trends have been observed, particularly across the Intermontane boreal forest, where temperatures have increased by approximately 0.37 to 0.39 °C decade^−1^. The Alaska range transition, Pacific mountain transition, and Coastal mountains transition have warmed between 0.28 to 0.29 °C decade^−1^^58,61^. Boreal forests are widespread across Alaska, bounded in the north by the Brooks Range and in the south by maritime Coastal rainforests and Aleutian meadows (e.g., Fig. 1). Boreal forest uplands and lowlands are underlain by discontinuous permafrost, where uplands are dominated by black spruce and white spruce and lowlands by fens, bogs, and marshes. The maritime ecoregions are located along the southern coast of Alaska and include a mix of discontinuous, sporadic, and isolated permafrost. These coastal ecoregions are characterized by forested landscapes with warm and wet climates that exhibit relatively limited annual temperature variation. The southern maritime ecoregions have experienced relatively lower rates of warming (i.e., 0.22–0.34 °C decade^−1^), compared to polar and boreal ecoregions. Precipitation in the Aleutian meadows increased by about 1.0% decade^−1^, while no significant change has been observed in other southern ecoregions.

### Historical peat-fire dynamics

Alaska statewide fire history perimeters, covering the period from 1950 to 2022, were downloaded from the Alaska Interagency Coordination Center (AICC)^35^. While this dataset is the most comprehensive fire history available for Alaska, the size of the burn scars delineated from satellite imagery (via*.*, spatial resolution < 30 m) decreased over time. For example, the minimum delineated fire area decreased from ~ 400 ha before 1987–40 ha between 1987 and 2015, and to 4 ha thereafter. To ensure consistency across our time-series, we included only fires with an area greater than 400 ha in our analysis. Using all available AICC data, we estimate fires smaller than 400 ha represent ~ 19,500 ha yr^−1^ or 0.17% of the annual total area burned. Additionally, we focused on fire history data after 1985 onwards, due to the increased reliability and frequency of satellite observations (i.e., Landsat), which minimizes the likelihood of missed burn scars.

We used the AICC data to estimate the spatial patterns and temporal trends of peatland areas burned across ecoregions of Alaska (Supplemental Fig. 3). The newly developed peatland map estimates that ~ 7.3% (110,133 km^2^) of Alaska’s land area consists of peatlands^31,33^. The peatland distribution varied across the ecoregions: the boreal ecoregions contain the largest area of peatlands, with 69,783 km^2^, representing 10.4% of the total ecoregion area. In the polar ecoregions, peatlands cover 26,842 km^2^, accounting for 4.6% of the total ecoregion area. The maritime ecoregions include 13,506 km^2^ of peatlands, which represents 5.3% of the total ecoregion area.

Although various peatland definitions and classifications exist^62^, our peatland products explicitly consider organic layer thicknesses greater than 40 cm as peatlands^31,33^. Using both our peatland map and AICC fire history data, we calculated two key metrics to assess the impact of wildfires on peatlands: (1) the annual total peatland area burned and (2) the proportion of peatlands burned. These peat-fire metrics were computed by summing the area of pixels identified as peatlands within each fire scar to estimate the total peatland area burned, which was divided by the total area of the fire scar to estimate the proportion of peatlands burned. The proportion of peatlands burned is different from the total area of peatlands burned (e.g., Supplemental Fig. 2), as we use it as a metric to describe the susceptibility of peatlands to wildfire. For example, an increase in this metric over time would suggest that wildfires are consuming a higher proportion of peatlands as fuels. While recognizing that wildfires do not uniformly burn all terrain within fire scar boundaries, our analysis assumes that all pixels within the AICC-derived fire perimeters burned. We acknowledge that this assumption may lead to an overestimation of the peat-fire area. However, supported by some studies^63^, our aim with this analysis was to provide a first-order estimate of the peatland area impacted by fire, as knowledge of peat-fire dynamics at this scale is currently limited^6,19,40^.

### Peat-fire modeling

To determine the statewide factors influencing the annual total peatland area burned and the proportion of peatlands burned within fire scars (i.e., metric describing the susceptibility of peatlands to fire), we developed two robust ensemble models using a combination of boosting, bagging, and stacking algorithms. Ensemble machine learning methods have proven to be highly effective predictive models^64–66^, offering a robust framework that minimizes the weaknesses and maximizes the strengths of individual machine learning models. The ensemble approach involves training multiple base models on the same training dataset, which are then used for prediction. A meta-learner is subsequently trained on the predictions of these base models, enabling it to adaptively improve overall performance. By aggregating the model predictions, assigning weighted averages based on model specific performance metrics, and correcting biases or inconsistencies during the process of model stacking, the ensemble method often produces more robust and accurate predictions compared to individual models. However, as fewer peat-fire observations are available within each ecoregion, we used the single best performing base machine learning model to predict peat-fire metrics by ecoregion.

The base models selected in the ensembles included boosting models (i.e., gradient boosting machines, extreme gradient boosting with a linear function and decision trees as base learners) and bagging models (i.e., random forests and decision trees), each offering strengths in handling non-normal data and multicollinearity among predictors (see Supplemental Table 1 for hyperparameters for base models). Gradient boosting machines and extreme gradient boosting (xgb) models are effective at capturing complex relationships and interactions in the data by sequentially fitting a base model to minimize a loss function. They are also capable of handling missing data and/or outliers, and less prone to overfitting. Gradient boosting tends to prioritize predictors with higher variable importance, potentially overlooking those with similar predictive power, while xgb addresses multicollinearity by randomly sub-setting predictors for each tree, similar to random forests. Random forest models use an ensemble of decision trees to reduce noise in the dataset, performing well with high-dimensional data and interactions, and are robust against overfitting. Decision tree models can effectively model non-linear relationships and interactions between variables, are robust to outliers, and missing values, and can handle various non-normal data types. These simpler models are robust to multicollinearity, as they partition the feature space recursively, selecting node splits based on independent predictors. However, decision trees are somewhat limited in their ability to capture complex relationships compared to more sophisticated boosting or bagging models like gradient boosting machines or random forests. Although these base models are robust to multicollinearity among predictors, we nevertheless implemented a multicollinearity analysis (i.e., Spearman’s pairwise correlations), where correlations greater than 0.7 or less than -0.7 were an indicator of multicollinearity among predictors.

We selected a range of potential predictors to provide critical information related to the flammability, dryness, and energy exchange conditions that influence peat-fire susceptibility^61^ for peatland burn metrics (i.e., total peatland area burned and proportional peatland area burned) from the European Centre for Medium-Range Weather Forecasts (ECMWF) ERA5-Land reanalysis dataset. Data for each predictor was aggregated daily and summarized annually. Mean annual air temperature (2 m above the land surface, °C) regulates soil thermal dynamics, promotes peat desiccation, and extends the fire season, increasing the window for ignition. As soil moisture influences both surface and subsurface fuel availability and flammability, we included the maximum volumetric soil moisture at layer 1 (integrated between 0 and 7 cm depths) and layer 4 (integrated between 100 and 289 cm depths; expressed as the volume fraction of water, representing commonly observed boreal peat depths^67^). Summer evapotranspiration reflects the net water loss from soils and vegetation during the warm season, contributing to drying and elevated fire risk, therefore we included the maximum summer evapotranspiration (daily maximum evaporation from vegetation transpiration; meters in water equivalent). In addition, as annual evaporation captures the cumulative moisture loss throughout the year; where high rates can lower water tables, desiccate peat, and create conditions conducive to combustion, we included the annual sum of evaporation (accumulated amount of water evaporated from the Earth’s surface; meters in water equivalent). Total annual precipitation (accumulated sum of rain and snowfall, meters) serves as the primary water input for peatland hydrology, where low annual totals of both rain and snow can result in long-term water deficits. Lastly, we included annual sum of latent heat flux (exchange of latent heat with the surface through turbulent diffusion; J/m^2^) and annual sum of sensible heat flux (transfer of heat between the Earth’s surface and the atmosphere through turbulent air motion; J/m^2^) as latent heat flux represents vegetation water use and fuel desiccation, signaling drought stress, while sensible heat flux indicates surface warming that can increase ignition potential.

Although the base models used in ensembles are robust to multicollinearity, we assessed collinearity among variables and removed redundant variables as appropriate (e.g., only a single layer of maximum volumetric soil moisture is used in each ensemble model). Both the statewide ensemble and ecoregion-specific models (i.e., random forest) used the same suite of predictors, with one exception, when modeling the proportion of peatlands burned, multicollinearity analysis (i.e., pairwise correlations) required different soil moisture variables by ecoregion. Specifically, boreal ecoregions incorporated maximum volumetric soil moisture from layer 4, while polar and maritime ecoregions used maximum volumetric soil moisture from layer 1.

Meta-learners combined predictions from four out of five base machine learning models to construct ensemble models, which included random forests, decision trees, and xgb with a linear function and decision trees as base learners. We rigorously validated the performance of these ensemble models using various metrics, including cross-validation, holdout validation, variable importances, ensemble-specific performance metrics, and comparisons with baseline models. Leave-one-out ten-fold cross-validation R-squares were implemented on all models. The datasets were divided into training and testing sets using a 70:30 split for independent testing set during holdout validation. Additional performance metrics, such as root mean squared error (RMSE) and mean absolute error (MAE) were employed to determine the highest performing base or ensemble model. Model interpretability was achieved using feature/variable importance analysis used to determine the most influential predictors across both ensemble and base and partial dependency plots of base models, used to interpret the models marginal effects of a predictor on model performance^68^.

Model uncertainties were assessed using two approaches: prediction uncertainty within the ensemble and bootstrap resampling. Ensemble prediction uncertainty was quantified by calculating the mean and standard deviation of predictions across all base models in the ensemble, providing a measure of variability among the models. Bootstrap resampling was applied to ecoregion-specific random forest models, where the model was repeatedly trained on random subsets of the data and evaluated on a test set (70:30 split). This process captured variability in predictions and was used to calculate 95% prediction intervals (lower and upper bounds). These methods provided a comprehensive evaluation of uncertainty associated with data variability, data limitations, and differences among models. All ensemble models were developed in R version 4.3.2 using the caret, caretEnsemble, randomForest, gbm, and xgboost packages, with uncertainty estimates computed using caretEnsemble and boot packages.

### Projected peat-fire dynamics

To predict the likely trajectory of peat-fire metrics by the end of the century, we used our ensemble models including all ecoregions and time-periods to estimate both historical (1970–1999) and projected (2090–2099) total peatland area burned and the proportion of peatlands burned across Alaska (Supplemental Fig. 3). Historical climate data was obtained from ERA5-Land reanalysis datasets^69^to represent a thirty-year peat-fire baseline, while the projected climate data was obtained from the top-5 performing Coupled Model Intercomparison Project, version 5 (CMIP5) models for Alaska^36^. The 5-model composite mean included the (1) MRI-CGCM3, (2) GISS-E2-R, (3) GFDL-CM3, (4) IPSL-CM5A-LR, and (5) NCAR-CCSM4, which were forced using representative concentration pathways (RCPs) 4.5 (low-emission), 6.0 (mid-range), and 8.5 (high-emission). These RCPs are climate change scenarios used to project future greenhouse gas concentrations^70^. However, as future CMIP5 model forcing data was limited to mean annual temperature and precipitation^36^, we estimated the seven other peat-fire model predictors (e.g., volumetric soil moisture, maximum evaporation) used in ensemble models to predict peat-fire activity (i.e., total and proportion of peatland area burned) not directly provided by CMIP5 products. We derived the strongest linear relationships between historical ERA5-Land temperature or precipitation and each predictor and applied these relationships to CMIP5 projections. Linear relationships with mean annual temperature included volumetric soil moisture (R^2^ = 0.43, sum of evapotranspiration from vegetation (R^2^ = 0.39), sum of latent heat flux (R^2^ = 0.73), sum of evaporation (R^2^ = 0.71), maximum potential evapotranspiration (R^2^ = 0.34), and the maximum evaporation (R^2^ = 0.51). We simulated the area of burned peatlands and the proportion of burned peatlands using statewide ensemble models with downscaled gridded climate and environmental parameters^36^, for 2090–2099 using the 5-model mean for three RCPs (4.5, 6.0, and 8.5) and for 1970–1999 using ERA5-Land reanalysis data used to estimate the potential future change across Alaska.

## Supplementary Information

Below is the link to the electronic supplementary material.

Supplementary Material 1

## Data Availability

Datasets used in this analysis are described and archived as follows: Peatland extent data of Alaska is published in the Arctic Data Center and as a data descriptor^31,33^, Alaska statewide fire history perimeters are provided by the AICC^35^, and climate datasets are provided by ERA-5 climate reanalysis datasets^69^, and CMIP5 models for Alaska^36,71^. For any related data or code requests, please contact Mark J. Lara (mjlara@illinois.edu).
