# Supplementary material for: Peatland fires in Alaska will double by the end of the century
Source: Sci Rep. 2025 Oct 14;15:35874. doi: 10.1038/s41598-025-19682-4 (PMC12521555; doi:10.1038/s41598-025-19682-4)
Supplement: Supplementary file 1 — Supplementary Material 1 [file 41598_2025_19682_MOESM1_ESM.docx]

**Supplementary Materials**

**for**

**Peatland fires in Alaska will double by the end of the Century**

Mark Jason Lara^1,2*^, Roger Michaelides^3^, Duncan Anderson^2^, Wenqu Chen^2^, Emma Catherine Hall^2^, Caroline Ludden^1^, Aiden Isaac Gittler Schore^1^, Umakant Mishra^4^, Sarah Nicole Scott^5^


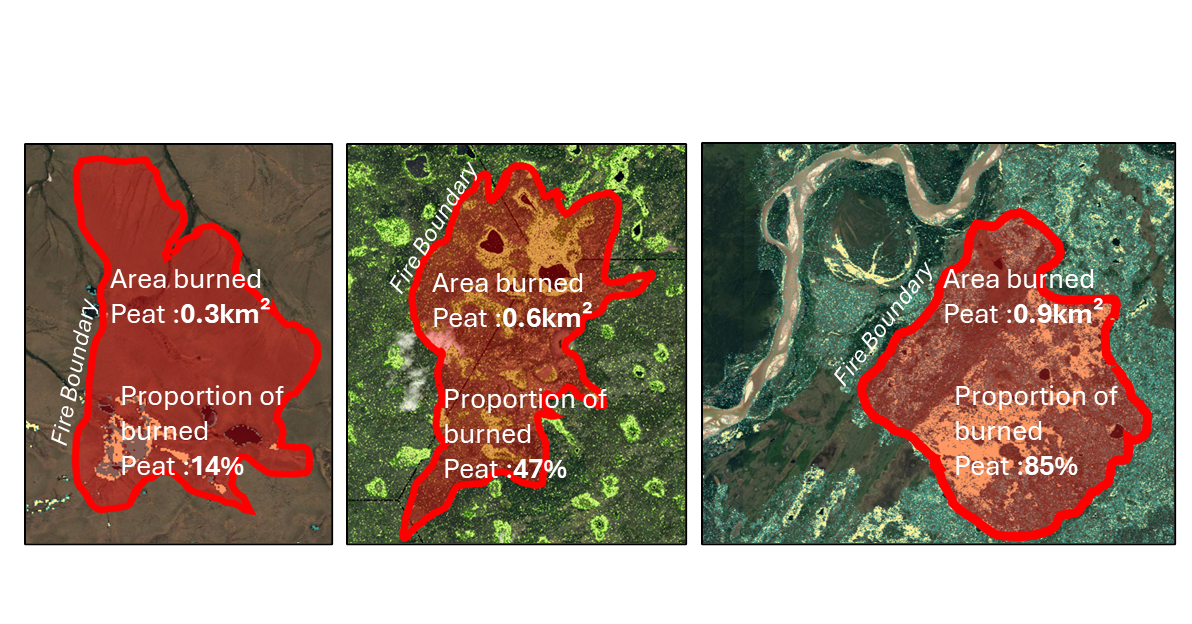


Supplemental Figure 1: Examples of the annual total peatland area burned and the proportion of peatlands burned. Total peatlands burned describe the total area of peatlands within a fire scar that burned, whereas the proportions of peatlands burned, describe the percentage of peatlands within the fire scar that burned. Example illustrates how the total area of peatlands burned may be similar, yet the proportion of peatlands burned can differ. ArcGIS version 10.8.1 (<https://www.esri.com/>) was used to create and visualize vector layers, overlaid on satellite images obtained from © 2015-2022 Maxar Technologies (<https://www.maxar.com/>).


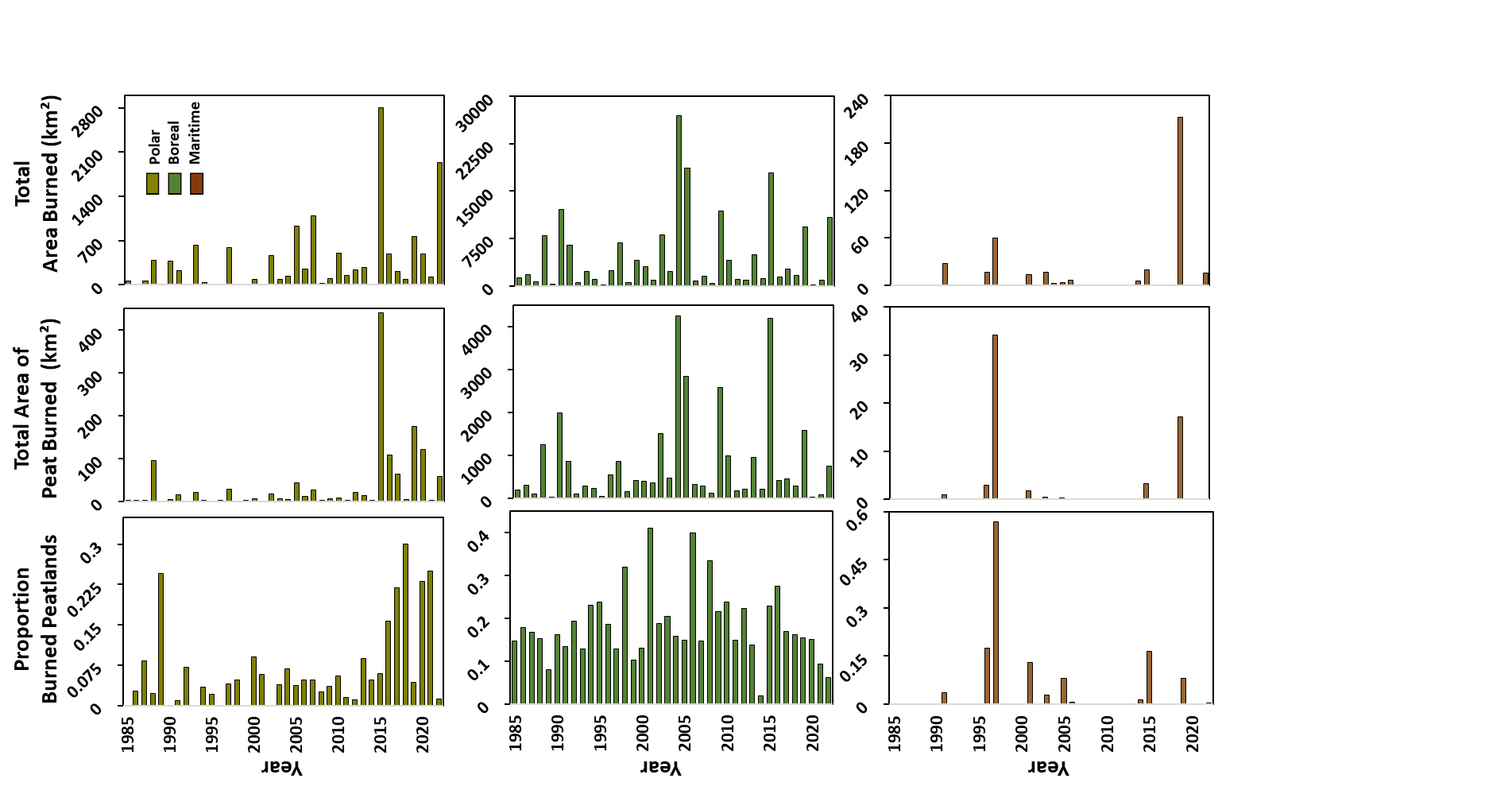


*Supplemental Figure 2: Annual fire metrics for ecoregions of Alaska. The proportion of peatlands burned (left column), total area of peatlands burned (center column), and the total annual area burned (right column) are described for Polar (top row), Boreal (center row), and Maritime (bottom row) ecoregions, respectively.*

*
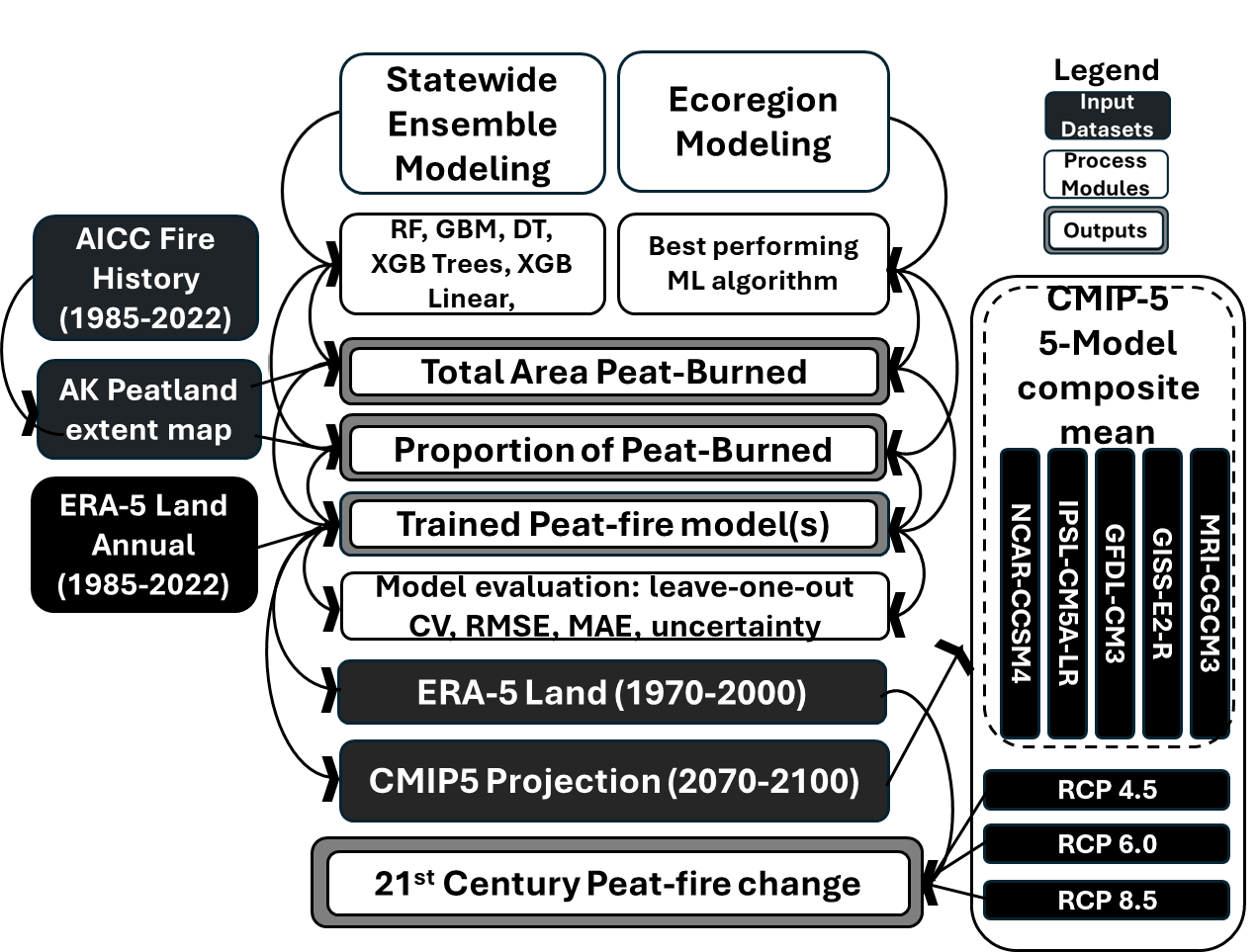
*

Supplemental Figure 3: Ensemble and ecoregion-specific modeling workflow.

Supplemental Table 1: Hyperparameters used in base models included in ensemble models.

| **Model** | **Hyperparameters** | ***Values*** |
| --- | --- | --- |
| RF | mtry | mtry = 2 |
| DT | No tunable parameters |  |
| XGB Linear | learning rate, lambda, alpha, nrounds | learning rate = 0.3, lambda = 0.1, alpha = 0.1, nrounds = 50 |
| XGB Tree | learning rate, gamma, max depth, fraccolsample, min_child_weight, subsample, nrounds | learning rate = 0.4, gamma = 0, max_depth = 3, fraccolsample = 0.8, min_child_weight = 1, subsample = 0.75, nrounds = 150 |
| GBM | trees, interaction.depth, shrinkage, minobs | trees = 50, interaction.depth = 3, shrinkage = 0.1 and minobs = 10 |
|  |  |  |

Supplemental Table 2: Model performance metrics for total peatland area burned and proportion of peatlands burned. Models include random forests (RF), decision trees (DT), extreme gradient boosting linear and tree (XGB Linear, XGB Tree), gradient boosting machine (GBM), and stacked ensemble model (Ensemble).

|  | **Total peatland area burned** | | | **Proportion of peatlands burned** | | |
| --- | --- | --- | --- | --- | --- | --- |
| **Model** | ***R*²** | ***RMSE*** | ***MAE*** | ***R*²** | ***RMSE*** | ***MAE*** |
| RF | 0.72 | 408.4 | 216.4 | 0.66 | 0.075 | 0.054 |
| DT | 0.76 | 487.4 | 289.9 | 0.29 | 0.079 | 0.061 |
| XGB Linear | 0.68 | 320.6 | 180.2 | 0.28 | 0.103 | 0.074 |
| XGB Tree | 0.74 | 394.7 | 245.6 | 0.34 | 0.079 | 0.061 |
| GBM | 0.79 | 512.1 | 317.5 | 0.49 | 0.074 | 0.054 |
| *Ensemble* | *0.89* | *277.4* | *117.7* | *0.71* | *0.062* | *0.042* |

Supplemental Table 3: Variable importance values for ecoregion specific random forest models. Importance values are ranked from most important to least important across both proportion of peatlands burned and total peatland area burned. NA indicates values that were not included in random forests. See methods for full description of variables.

|  | **Proportion of peatlands burned** | | | **Total peatland area burned** | | |
| --- | --- | --- | --- | --- | --- | --- |
| **Variable Importance** | ***Polar*** | ***Boreal*** | ***Maritime*** | ***Polar*** | ***Boreal*** | ***Maritime*** |
| Temperature | 60.7 | 100.0 | 100.0 | 66.5 | 19.4 | 100.0 |
| Latent Flux | 46.4 | 74.6 | 64.6 | 100.0 | 14.4 | 43.8 |
| Evapo Sum | 100.0 | 61.6 | 7.8 | 93.2 | 26.1 | 6.4 |
| ET veg Sum | 27.7 | 27.4 | 0.0 | 46.7 | 100.0 | 0.0 |
| Precipitation | 2.8 | 0.0 | 48.6 | 51.1 | 0.0 | 41.9 |
| Soil Moisture 4 | 0.0 | *NA* | 25.3 | *NA* | 94.1 | 15.8 |
| ET max | 6.1 | 60.2 | 4.5 | 21.0 | 5.9 | 25.7 |
| Sensible Flux | 2.7 | 25.5 | 21.3 | 27.4 | 0.2 | 15.0 |
| Soil Moisture 1 | *NA* | 69.26 | *NA* | 0 | *NA* | *NA* |


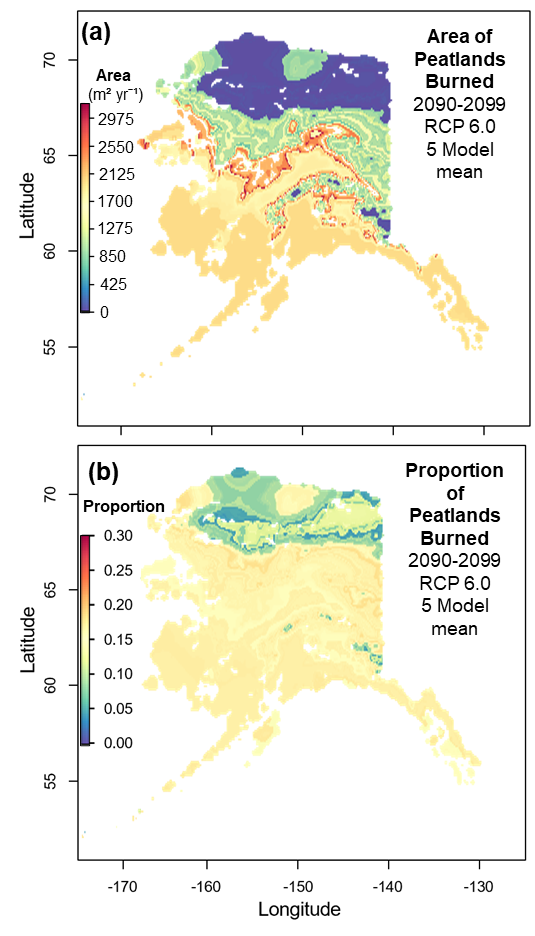


Supplemental Figure 4: Ensemble model projected patterns of peat-fire metrics across Alaska. Climate forcing data (2090-2099 mean) were derived from the top-5 performing CMIP5 models for Alaska^36^ for representative concentration pathway 6.0. Peat-fire models and associated geospatial layers were generated in R version 4.3.2 (<https://www.r-project.org/>) and visualized in ArcGIS version 10.8.1 (<https://www.esri.com/>).

Supplemental Table 4: Historical and Projected means, change, and percent change in peat-fire metrics.

|  |  | **Total peatland area burned** | | | | **Proportion of peatlands burned** | | | |
| --- | --- | --- | --- | --- | --- | --- | --- | --- | --- |
| **Simulation** | **Metric** | ***Statewide*** | ***Polar*** | ***Boreal*** | ***Maritime*** | ***Statewide*** | ***Polar*** | ***Boreal*** | ***Maritime*** |
| Historical | Mean | 397.4 | 320.5 | 456.5 | 433.6 | 0.104 | 0.090 | 0.123 | 0.087 |
| Projected RCP 4.5 | Mean | 641.2 | 400.7 | 752.7 | 877.2 | 0.142 | 0.120 | 0.159 | 0.153 |
| Projected RCP 6.0 | Mean | 640.1 | 418.9 | 733.7 | 879.9 | 0.144 | 0.123 | 0.159 | 0.154 |
| Projected RCP 8.0 | Mean | 877.1 | 848.9 | 889.5 | 894.4 | 0.158 | 0.155 | 0.164 | 0.152 |
| Projected RCP 4.5 | Change | 243.8 | 80.2 | 296.2 | 443.6 | 0.038 | 0.029 | 0.035 | 0.066 |
| Projected RCP 6.0 | Change | 242.7 | 98.4 | 277.2 | 446.3 | 0.040 | 0.033 | 0.035 | 0.067 |
| Projected RCP 8.0 | Change | 479.7 | 528.4 | 433.0 | 460.8 | 0.054 | 0.064 | 0.040 | 0.065 |
| Projected RCP 4.5 | Percent change | 61.3% | 25.0% | 64.9% | 102.3% | 36.5% | 32.4% | 28.6% | 75.8% |
| Projected RCP 6.0 | Percent change | 61.1% | 30.7% | 60.7% | 102.9% | 38.5% | 36.5% | 28.6% | 77.0% |
| Projected RCP 8.0 | Percent change | 120.7% | 164.9% | 94.9% | 106.3% | 51.9% | 71.0% | 32.8% | 74.5% |
|  |  |  |  |  |  |  |  |  |  |
